# Femtosecond laser 3D nanoprinting using inorganic nano-building blocks

**DOI:** 10.1016/j.xinn.2024.100772

**Published:** 2024-12-24

**Authors:** Yi-Ke Sun, Yu-Qing Liu, Qi-Dai Chen, Yong-Lai Zhang

**Affiliations:** 1 State Key Laboratory of Integrated Optoelectronics, College of Electronic Science and Engineering, Jilin University, Changchun 130012, China; 2 Center for Advanced Optoelectronic Functional Materials Research, Key Laboratory for UV Emitting Materials and Technology of Ministry of Education, National Demonstration Center for Experimental Physics Education, Northeast Normal University, Changchun 130024, China

## Main text

**Figure 1:**
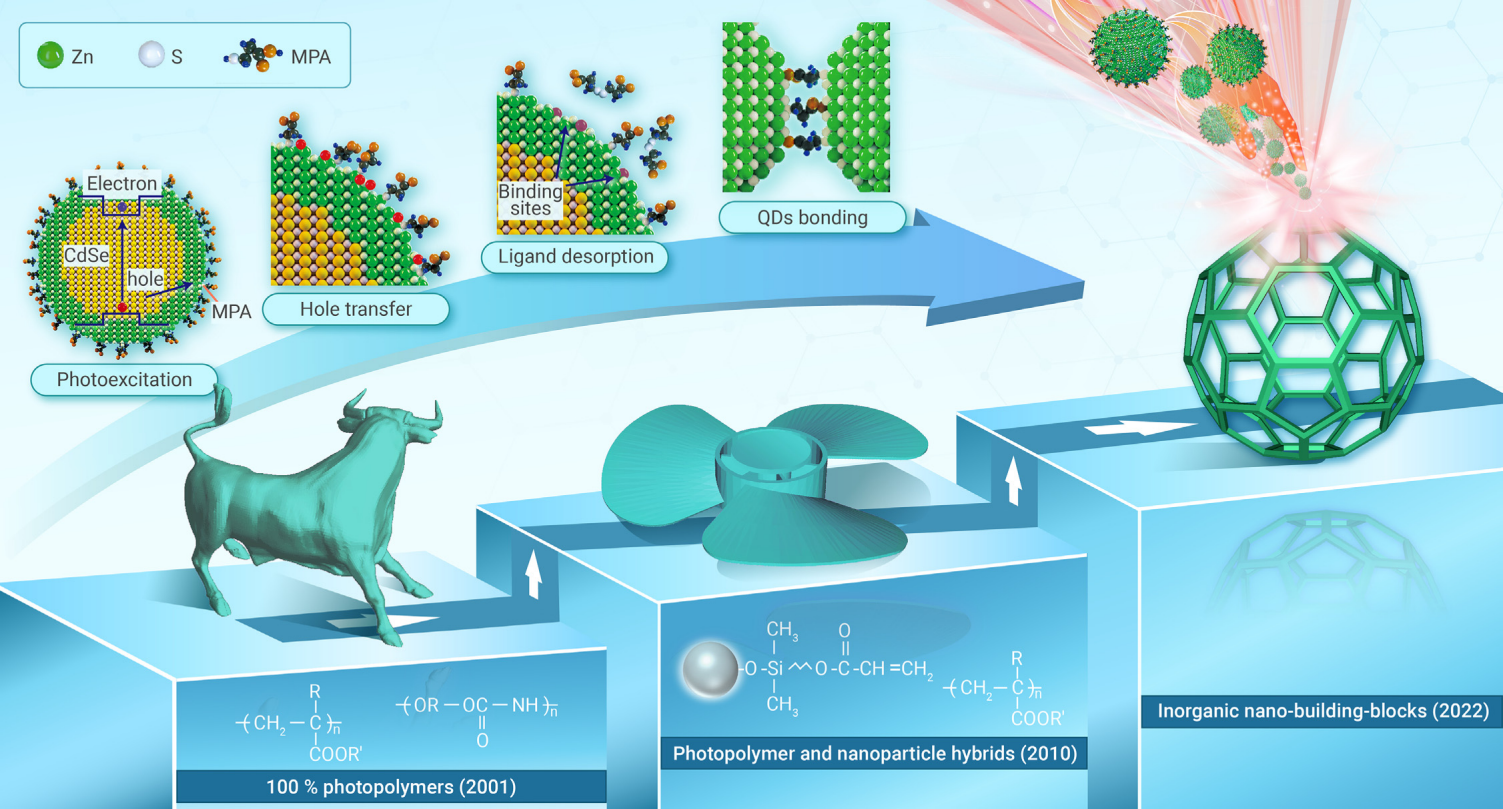
Progress in femtosecond laser TPP fabrication from organic photoresists and inorganic/organic hybrids to pure inorganic NBBs The inset shows the mechanism of femtosecond laser nanoprinting of ZnS QDs. Reproduced with permission.^4^ Copyright 2022, AAAS.

Since the 1990s, femtosecond laser two-photon absorption (TPA), which enables point-by-point photopolymerization, has been a tool for making three-dimensional (3D) microstructures. Approximately 10 years later, Kawata et al. improved the spatial resolution of TPA fabrication to ∼120 nm, far beyond the diffraction limit of the 780 nm laser source.^1^ The essential mechanism of this nanoscale fabrication lies in the nonlinear effect, in which TPA-induced photopolymerization occurs only in the vicinity of the focal spot. As a landmark symbol of this field, “microbull” sculptures, ∼10 μm in length and 7 μm in height, have been produced using a commercially available resin that consists mainly of urethane acrylate monomers, oligomers, and photoinitiators (Figure 1, left inset). Currently, both spatial resolution and fabrication efficiency have been significantly promoted, and TPA fabrication has emerged as a sophisticated 3D nanoprinting protocol. However, the available materials for TPA fabrication are largely limited to photocurable resins, and femtosecond laser 3D printing of functional nanomaterials beyond polymers remains a challenging task.

To extend printable materials to inorganic substances, alternative strategies have been developed for femtosecond laser 3D nanoprinting. The first is the conformal deposition of inorganic materials on 3D polymer structures, which results in the formation of 3D inorganic shells. The second strategy involves mixing inorganic nanomaterials with photoresists, forming photocurable organic-inorganic nanohybrids for direct 3D nanoprinting. As a typical example, Xia et al. developed a ferrofluid resin composed of methacrylate group-modified Fe_3_O_4_ nanoparticles and photoresists, by which magnetic micromachines capable of remote manipulation have been fabricated (Figure 1, middle inset).^2^ For these hybrid precursors, photopolymers mainly contribute to 3D structuring, working as structural components, whereas inorganic nanomaterials provide additional functionalities, such as magnetism, fluorescence, conductivity, and antibacterial properties, serving as functional components. In general, special attention should be given to the trade-off between structural and functional components, especially those that require postsintering to remove organic components. Theoretically, direct printing of inorganic materials might be a solution to this issue. Therefore, novel photochemical/photophysical schemes beyond photopolymerization, such as the photoreduction of metal ions for 3D metal nanoprinting, have been successfully developed. Nevertheless, unlike photopolymerization, the formation of metallic structures may consume many ions around the focal spot. The lack of an ion source constitutes the main obstacle to the formation of inorganic 3D microstructures.

Currently, there is an urgent need to develop emerging 3D nanoprinting technologies based on novel functional materials.^3^ Considering their superior electronic, optical, and magnetic properties, inorganic nanoparticles are ideal nano-building blocks (NBBs) for manufacturing functional devices via various bottom-up and top-down approaches. For example, optical lithography or other photodynamic printing techniques, such as bubble printing, have already demonstrated the possibility of fabricating arbitrary inorganic patterns with high resolution. However, in most cases, the as-obtained structures are limited to 2D patterns, although some of them are created on nonplanar substrates. The combination of inorganic NBBs with additive manufacturing technologies, such as femtosecond 3D nanoprinting, can address these limitations well. To reach this goal, the key challenge lies in the programmable assembly of NBBs into well-defined 3D structures, in which both the trapping force for inorganic nanoparticles and their interactions with each other should be taken into account.

Recently, Liu et al. proposed a photoexcitation-induced chemical bonding (PEB) approach that enables femtosecond direct writing of inorganic 3D microstructures using semiconductor quantum dots (QDs) as NBBs (Figure 1).^4^ This technique utilizes optically controlled detachment and rebinding of surface ligands to form interparticle chemical bonds, enabling the subsequent 3D printing process. Taking CdSe/ZnS QDs capped with 3-mercaptopropionic acid (MPA) as an example, the highest occupied molecular orbital of MPA molecules is located above the maximum valence band of CdSe. The photoexcitation-generated holes transfer to the surface and trigger the desorption of MPA ligands, leaving unoccupied active sites for interparticle bonding between nearby QDs. By programming the pathway of the focused femtosecond laser inside the QD solution, the dispersed QDs can be printed into densely packed 3D structures with sub-100 nm resolution via the PEB process. This technique has also been applied to CdSe/ZnS QDs of different colors and other MPA-capped QDs and silver nanoparticles. Nevertheless, it still relies on specific combinations of NBBs and surface ligands, thus limiting the applicability of other inorganic nanomaterials.

To further enrich the library of printable materials, the same group developed a material-independent technique for 3D printing inorganic nanomaterials via photochemical bonding.^5^ Inspired by atom-scale matter organization, inorganic NBBs capped with native ligands have been considered artificial “atoms,” and a small number of photosensitive bisazide molecules have been employed as artificial “chemical bonds.” Under femtosecond laser irradiation, the bisazide molecules generate reactive nitrene radicals at both ends, and C–H insertion with QD ligands occurs. Ligand-linking events first take place on the same QD, which reduces interparticle repulsion and facilitates chemical bonding among nearby QDs. In this way, inorganic 3D microstructures can be created by scanning the focal spot. The printing chemistry was further validated by the formation of C–N bonds in the printed structures, as well as comparisons between photoacid molecules with noncrosslinking capabilities.

Given the generality of nitrene-mediated chemical bonding, this strategy has been adapted to a broad spectrum of inorganic nanomaterials, as exemplified by more than 10 semiconductors, metal oxides, metals, and their mixtures. Various inorganic NBBs can be assembled into complex 3D structures, multicomponent structures, and heterogeneous structures. The material versatility and printing capability make it possible to produce diverse functional devices on demand, which endows the printed microstructures with new functionalities. For example, the semiconductor nanohelical arrays exhibit broadband visible and near-infrared chiroptical responses with a high anisotropic factor of 0.24, which is ∼20 times greater than that of self-assembled chiral structures. Owing to efficient bonding chemistry, femtosecond laser 3D nanoprinting of inorganic NBBs can produce high-purity inorganic structures with a large inorganic mass fraction greater than 90%. The remaining organic components originate from the native surface ligands and a small amount of bisazide additives (as low as ∼0.2 wt %). In addition to their high purity, the printed materials can retain the intrinsic properties of the QDs, such as size-dependent absorption and photoluminescence properties. Interestingly, the femtosecond laser 3D printing of inorganic NBBs is similar to nacre biomineralization. Although the organic fraction is low, the strong C‒N covalent bonds between NBBs contribute to the high mechanical robustness of the as-printed microstructures. The micropillars composed of QDs have both high compressive strength (>1 GPa) and high fracture strain (∼55%).

In summary, recent advances in femtosecond laser 3D nanoprinting have proven the possibility of 3D microstructuring using inorganic NBBs, in which new photochemical schemes beyond TPA have been successfully developed. The breakthrough in material applicability with respect to femtosecond laser fabrication may bridge the gap between a dazzling array of inorganic nanomaterials and various functional microdevices. Nevertheless, femtosecond laser 3D nanoprinting using inorganic NBBs is still at an early stage, and a series of challenges exist to the further progression of this promising nanotechnology. For example, different inorganic nanomaterials are capped with different surface ligands; thus, the universality of this technology requires more experimental cases to confirm, and novel photochemical/photophysical schemes are highly desirable. In addition, the size dependence of the printable inorganic building blocks (from subnanometer nanoclusters to microscale particles) deserves more careful study. More importantly, future research on this topic should focus more on the development of novel functional devices for cutting-edge applications in different fields. We believe that with the continuous advancement of this nanotechnology, femtosecond laser 3D nanoprinting using inorganic NBBs may emerge as a nanoenabler to fabricate nanoscale functional devices that are otherwise not possible.

## Acknowledgments

This work was supported by the 10.13039/501100012166National Key R&D Program of China (no. 2022YFB4600400) and the 10.13039/501100001809National Natural Science Foundation of China (nos. T2325014, 62205174, and 623B2042). The funders had no role in the study design, data collection and analysis, decision to publish, or preparation of the manuscript.

## Declaration of interests

The authors declare that they have no competing interests.
